# New horizons in spine research: Disc biology, tissue engineering, biomechanics, translational, and clinical research

**DOI:** 10.1002/jsp2.1032

**Published:** 2018-08-29

**Authors:** Chitra L. Dahia, James C. Iatridis, Makarand V. Risbud

**Affiliations:** ^1^ Orthopedic Soft Tissue Research Program Hospital for Special Surgery New York New York; ^2^ Department of Cell and Development Biology Weill Cornell Medicine, Graduate School of Medical Sciences New York New York; ^3^ Leni & Peter W. May Department of Orthopaedics Icahn School of Medicine at Mount Sinai New York New York; ^4^ Department of Orthopaedic Surgery, Sidney Kimmel Medical College Thomas Jefferson University Philadelphia Pennsylvania

## 1 INTRODUCTION

Lower back and neck pain is now considered the top neurological disorder affecting over 540 million people worldwide, yet one that has no definitive treatments. The incidence of back pain increases with age, is higher in women than in men, and almost 80% people have at least one episode of back pain in their lives.^1^ While lower back pain is a multifactorial disorder, it is closely associated with intervertebral disc dysfunction.^2^ One of the strategies to treat back pain is to restore the structure and function of the intervertebral disc, which requires further research for a better understanding of these mechanisms.^3^, ^4^, ^5^ The gap in research directions that could enhance our fundamental understanding of the intervertebral disc, as well need for research funding was highlighted by the National Institute of Arthritis and Musculoskeletal and Skin Diseases with a Roundtable on the Role of Disc Degeneration in Neck and Back Pain.^6^ To advance research in different areas related to intervertebral disc and spine, scientists from around the globe gathered at the 4th International Orthopaedic Research Society and Philadelphia Spine Research Symposium (ORS/PSRS) was held at Lake Harmony, PA, USA from Oct 23rd to 27th, 2017.^7^ The Symposium was a 3.5 day meeting that included scientific presentations by internationally recognized spine and intervertebral disc scientists and open presentations in topic areas including: disc development and biology; disc pathology and pain; tissue engineering of the disc; disc mechanobiology; and animal models/ translational/ pre‐clinical studies. This ORS/PSRS Symposium provided a platform to learn about recent scientific advances, to discuss future research directions, and to foster collaborations aimed to advance disc related research further to develop novel disc therapies and build better outcomes for back pain treatments. This Special Issue is an outcome of the 4th International ORS/PSRS Spine Research Symposium. The papers compiled for this Special Issue include invited review articles collaboratively authored by invited faculty from scientific areas highlighted in the meeting, and also include original research from studies presented at the meeting that won podium and poster awards and by attendees of the meetings. This Special Issue is based on the scientific themes and sessions of the Symposium and provides a glimpse of some of the cutting‐edge research and international collaborations in the intervertebral disc research field.

## 2 COLLABORATIVE REVIEW ARTICLES ON SCIENTIFIC TOPICS OF THE MEETING

*Development and disc biology*: Seguin et al,^8^ reviewed the current research on the origin of the different components of the intervertebral disc including nucleus pulposus and annulus fibrosus. The current literature on role of different signaling pathways including sonic hedgehog (Shh), transforming growth factor beta (TGFβ), and transcription factors including Noto, Brachyury (also known as Bra or T), Sox −5, −6, −9, and Scleraxis during development and postnatal stages of intervertebral disc are reviewed. Also why this information is relevant for improving techniques for disc therapy is discussed. Finally, this review article discusses the recent findings on molecular heterogeneity in the nucleus pulpous cells during postnatal stages and implications for the development of strategies for disc regeneration.

*Disc tissue engineering*: Buckley et al 9 reviewed and discussed the current approaches in tissue engineering and regeneration to treat intervertebral disc disorders. This review also highlights the role of disc microenvironment and mechanical properties that may influence the success of the disc repair and regenerative strategies with description of some of the harsh microenvironment factors known to be factors in disc degeneration and aging that may affect the disc repair and regeneration strategies. Several of the current advances in tissue engineered material, mechanical, biological and clinical opportunities and constraints for clinical translation of regenerative therapies for disc treatment are also described.

*Disc mechanobiology*: Fearing et al 10 have reviewed the structure and biochemical composition of the intervertebral disc and how various mechanical forces including compression, tension, hydrostatic pressure and osmotic effects the disc cells and their function. This review also discusses the effects mediated by sub‐cellular mechanotransduction pathway on cell‐cell interaction, cell‐matrix interaction, and cytoskeletal remodeling. Also, the literature on the abnormal mechanobiology during aging and disc degeneration is discussed.

*Animal models/translational/pre‐clinical studies*: Thorpe et al 11 reviewed the hurdles in the commercialization of scientific findings and protecting intellectual property. This review discusses the steps and obstacles involved including the inception of a scientific idea, transition into preclinical model, and successful translation into clinical practice for the development of regenerative strategies for the intervertebral disc, as a potential treatment for back and neck pain. Emphasis is made on prior planning as well as communicating with the various parties including patient, payer and scientist involved in the process is critical for successful translation for research findings for improving the health and quality of life of patients.

### 2.1 Award‐winning research paper

*Optimized culture system for notochordal cell expansion with retention of the phenotype*: Humprey et al, describes an improved and streamlined technique to culture and expand notochordal cells for their potential use for disc treatment and regeneration.^12^ The potential of notochordal cells for therapy of disc disease has the potential for success. However, the limitation of the success of using notochordal cells is mainly due to the reduced yield of the notochordal cells, and retention of their molecular profile in culture. In their study Humprey et al first show that neonatal porcine notochordal cells are similar to human fetal notochordal cells. Next, the authors expanded the neonatal porcine notochordal cells in culture while retaining their molecular signatures since the expression of Brachyury (T), KRT8, KRT18, KRT19, and CD24 was maintained in these expanded cells. This study provides hope for notochordal cells in disc therapy.

### 2.2 Submission by meeting attendee

*A resorbable plating system for stabilization of intervertebral disc implant*: Total disc replacement or disc arthroplasty was thought to provide an alternative to the traditional care for therapy of the cervical disc degeneration. However, due to the onset of adjacent segment disease it was not successful. Total disc replacement using tissue‐engineered intervertebral disc (TE‐IVDs) plates that mimics the native disc provides a potential for biological alternatives to therapies for degenerative disc disorders. To restore the motion and improve the stability of the spine motion segments using TE‐IVDs, Mojica Santiago et al, have tested the potential of the bio‐resorbable plates using fixation system of 85:15 polylactic‐co‐glycolic acid plates and screws (Rapidsorb, Depuy Synthes Co. Johnson & Johnson, West Chester, PA) using canine cervical spines. The findings by Mojica Santiago et al, show that the plated segments partially restored motion segment stiffness compared to controls.^13^ Also, attachment of the resorbable plates prevented extrusion of the implant from the disc space. Hence, the potential of these plates to fully integrate into the host tissue provides hope for improving current approaches for disc arthroplasty.

## 3 CONCLUSIONS

This Special Issue provides a review of current advances in the different areas of intervertebral disc research including disc development, disc biology, tissue engineering of the disc, disc biomechanics, animal models, and how to successfully translate the research findings from the laboratory into clinical practice which are advanced with the additional submitted papers. Together, this Special Issue included novel techniques and concepts that are hoped to advance and inspire technologies that will lead to better outcomes and approaches for disc repair and the treatment of back and neck pain.

### Conflict of interest

None of the authors have any conflict of interests to disclose.
